# Turning over New Leaves

**Published:** 1888-12-08

**Authors:** 


### TURNING OVER NEW LEAVES.

[Books for Review should be sent to The Editor, The Lodge, Porchester Square, W.]

### Heroes of Every Day Life.*

MISS LANE'S book deals with those heroes on whose banners should be written "our duty is to save." But such heroes and heroines do not carry banners, and as a rule are by no means conscious of their heroic qualities. Their names are not often inscribed in the bead-roll of more enduring fame than that given by local newspapers, and Miss Lane confesses that the example she gives of simple bravery by no means exhaust the list of heroes of every day life, but are simply "sheaves from that rich harvest of golden deeds which yet remains to be gathered in." They are widely representative, men and women, old and young, but all members of the great English race which has set its adventurous foot on the utmost corners of the earth. It would be impossible in a brief notice to enumerate the names and deeds of all the noble men and women of whom Miss Lane tells us; but it is worth while to speak of two or three. First, we may select the dynamite heroes, Messrs. Cole and Cox, the policemen who carried out of Westminster Hall the case of smouldering dynamite, which, in a few more minutes, would have destroyed a historic building, and perhaps brought death or permanent injury to many innocent sightseers. In spite of the frank and sympathetic eulogy which Louis Stevenson gave them in the preface to "The Dynamiter," these two brave policemen and their quiet daring can hardly be said to have received adequate appreciation from the public; and it is not unfitting at this time, when so many reproaches are being hurled at "the force," to bring back to memory one out of the many brave deeds of "the boys in blue" who guard the public safety, on the whole, so well.

Another brave deed which is well-nigh forgotten is that of Alice Ayres, the maid-o-fall-work, who lost her life when her master's house and shop caught fire, but not before she had saved his three children. First she forced a featherbed through a narrow window, then plunged back through the smoke and flames to bring them one by one and fling them down on the bed that was held out to catch them below. After they were safe she jumped herself, but her eyes were dim and her brain reeling with the heat and the smoke. She did not leap far enough, struck against the sign above the shop door, and fell on the pavement. Her spine was broken, and she died two days afterwards in Guy's Hospital. To all comments and eulogies on her conduct her answer was a very simple one: "I tried to do my best."

Another woman who tried to do her best was the Australian girl, Grace Bussell, who—it sounds paradoxical, but is true none the less—saved the shipwrecked passengers of the steamer *Georgette*, on horseback. The vessel was stranded, and the boats that left her were overturned by the raging surf. The crew and passengers were being drowned within sight, almost within touch, of land, when Grace Bussell, a girl of sixteen, who was riding through the bush not far from the coast, accompanied by a native stockman, saw their plight. At once she rode down a steep cliff path at a headlong pace, and forcing her horse into the surf, rode out to the struggling creatures who were clinging to the overturned boat, and carried them to the shore. The stockman, who had at first stood aghast at his young mistress's daring, followed her, and shared in the work of rescue. Having got the shipwrecked crew to shore—a task that took four hours to accomplish—she rode to her home to make arrangements for their reception there. They were welcomed, fed, and clothed, but at a heavy cost to the Bussell family, for four days after they left Grace's mother died of brain fever, brought on by the fatigue she had undergone in providing for and attending to these uninvited guests, many of whom were suffering from the effects of their long exposure. Her last coherent words were, "Fetch them all, I can take them all in," showing that even when her brain was distraught, her heart was true to the claims of humanity.

It is hardly necessary to speak of Mrs. Fox, who ministered when wounded herself to the suffering soldiers in the Transvaal. It is not long since we recorded in our columns her death, and the military funeral which the Commander-in-Chief gave orders she should receive, because, as the colonel of the 94th said, "She died a soldier's death, as her fatal illness was the result of a wound received in action and aggravated by consequence of her noble self-devotion afterwards."

Other tales of courage there are, of rescues from sinking ships and stormy waters, of men taken from the depths of choking and burning mines, or even from the vile fumes of sewers. Miss Lane's style is occasionally verbose and effusive, but her matter is well worth reading. We must note, however, that Bret Harte is not the author of the ballad of Jim Bludso, who saw

"His duty, a dead sure thing,

And went for it there and then."

* "Heroes of Every Day Life": By Laura M. Lane, Author of "A Nineteenth Century Hero," etc., Cassell and Co. (Limited).

### The Law of Liberty.*

"THINK of that poor negro woman," says Dr. Whiton, "who bequeathed the savings of a life spent at the wash-tub to the Yale Theological Seminary, to be a fund for the education of indigent men of her own race to preach the Gospel of Christ. She lived by her wash-tub, but she did not live in it: she lived above it. . . Her outward life was common and low; but her inner life was dignified by an interest that made the struggle of her aspiring race her struggle, their achievements her achievements. . . Here we have found on one of the humblest levels of the modern world, a life of the commonest drudgery, filled with dignity and power by the same divine object that inspired Paul's life of tribulation with thanksgiving. . . The gill measure, the gallon measure: the laundress, the Apostle—are both full of the same inspiration." This story sounds the key-note to Dr. Whiton's sermon on the law of liberty, and it need not be doubted that the same strain of simple, but pleasing and effective, music runs through the book. "Thou shalt," or "thou shalt not," the writer thinks is a poor level for a man of any intellectual force and moral wholesomeness to attain to. "I will" do this, that, or the other good thing, not merely because it is my duty, but because it is my preference. I love the whole atmosphere of goodness, of neighbourliness, of sympathy with my fellows. This alone is worthy of Christian brains. A life of mere self-advancement and self-pleasing is not only morally poor, but intellectually stupid. The cow or the pig can rise to that level. The man should rise to as much higher an elevation as he is morally and intellectually superior to the cow and the pig. Many volumes of sermons are published, but not read. Anyone who desires a small volume of American discourses, which are both readable and reasonable, will do well to invest a few shillings with Dr. Whiton. The price of the book is not given, but it cannot be high.

* "The Law of Liberty": By J. Morris Whiton, LL.D. London: James Clarke and Co.

